# Human Vulnerability to Landslides

**DOI:** 10.1029/2020GH000287

**Published:** 2020-10-01

**Authors:** William Pollock, Joseph Wartman

**Affiliations:** ^1^ Department of Civil and Environmental Engineering University of Washington Seattle WA USA

**Keywords:** landslide, vulnerability, risk analysis, mortality, probability of death, disaster

## Abstract

Landslides pose a devastating threat to human health, killing thousands of people annually. Human vulnerability is a crucial element of landslide risk reduction, yet up until now, all methods for estimating the human consequences of landslides rely on subjective, expert judgment. Furthermore, these methods do not explore the underlying causes of mortality or inform strategies to reduce landslide risk. In light of these issues, we develop a data‐driven tool to estimate an individual's probability of death based on landslide intensity, which can be used directly in landslide risk assessment. We find that between inundation depths of approximately 1–6 m, human behavior is the primary driver of mortality. Landslide vulnerability is strongly correlated with the economic development of a region, but landslide losses are not stratified by gender and age to the degree of other natural hazards. We observe that relatively simple actions, such as moving to an upper floor or a prepared refuge space, increase the odds of survival by up to a factor of 12. Additionally, community‐scale hazard awareness programs and training for citizen first responders offer a potent means to maximize survival rates in landslides.

## Key Points

- Many modern estimates of human vulnerability to landslides rely on subjective judgment and ignore the role of human behavior
- We present a human vulnerability curve that links an individual's probability of death to landslide inundation depth
- We find that human behavior drives vulnerability at inundation depths of 0.9–5.9 m and suggest practical actions to reduce personal risk

## 1 Introduction

Due to their high velocity, large impact pressures, and the ability to run out long distances over flat terrain, rapid landslides are a particularly devastating threat to human health. From 2004–2016, landslides have caused an annual average of over 4,000 deaths worldwide, and in the United States alone, they are estimated to kill 25–50 people each year (Froude & Petley, 2018; Schuster & Highland, 2001). Understanding human vulnerability to landslides is essential for predicting and preventing human loss of life. While much scholarship has been devoted to quantifying the physical vulnerability of buildings to landslides, there is no comparable work that quantifies human vulnerability for use in risk assessment. The few methods that exist rely extensively on expert judgment and are not easily transferable (AGS, 2007; Corominas et al., 2014; Lee & Jones, 2014). Furthermore, these methods only inform the potential for human losses and do not explore the underlying causes of human mortality, leaving scientists, decision‐makers, emergency responders, and the public without evidence‐based strategies for maximizing survival rates in landslides (Kennedy et al., 2015). In this paper, we address this void by (1) presenting a new landslide fatality data set; (2) creating an empirical human vulnerability relationship to estimate an individual's probability of death; (3) detailing the human characteristics, behaviors, and settings that contribute to landslide mortality; and (4) proposing a suite of evidence‐based, actionable strategies to minimize personal landslide risk.

## 2 Background

### 2.1 Landslide Modes

The term “landslide” has many meanings in academic and colloquial usage. In this work, we focus on rapid landslides, having velocities of >5 m/s (Cruden & Varnes, 1996), as these pose the greatest threat to human life (Petley, 2010). All the events considered here fall within the categories of debris flow, flowslide, rock/debris avalanche, or debris slide (Hungr et al., 2014), with the majority being highly fluidized, channelized debris flows spreading onto populated depositional fans.

### 2.2 Vulnerability

Vulnerability is *the potential to suffer harm from a human perspective*. The natural sciences focus on *physical vulnerability*, which quantitatively describes the degree or probability of tangible damage, injuries, or deaths on a scale from zero (none) to one (complete). Physical vulnerability is a fundamental component of risk analysis (Fell et al., 2005). A recent emphasis on vulnerability—rather than hazard—as the primary driver in environmental disasters has prompted numerous efforts to quantify human and infrastructural vulnerability to different natural hazards such as snow avalanches (Barbolini et al., 2004), tsunamis (Reese et al., 2007), floods (Milanesi et al., 2015; HR Wallingford, 2006), hurricanes (Pita et al., 2011), and earthquakes (Spence et al., 2008).

### 2.3 Human Vulnerability to Landslides

The physical vulnerability of infrastructure to landslides has been the subject of an emerging body of data‐driven studies and practical tools for practitioners (Fuchs, Keiler, et al., 2019; Massey et al., 2019; Papathoma‐Kohle et al., 2015). Although infrastructural losses are of secondary importance to the risk to humans themselves, research investigating the vulnerability of people to landslides is rare (Glade, 2003; Lin et al., 2017; Massey et al., 2019). The reasons for this are manifold. Human casualties in landslides are often related to the collapse of occupied buildings and thus are indirect, a function of structural vulnerability (Jakob et al., 2012). Human vulnerability also depends on human behavior, including factors that are difficult to measure, such as prior knowledge of hazards, situational awareness, and decision‐making capability (Eidsvig et al., 2014). Human data are ephemeral and challenging to collect in the chaotic aftermath of a disaster. While damaged buildings are measurable weeks or months after an event, the people involved will often have relocated, been dispersed to hospitals, or be dead. Finally, ethical approval is required for human subjects research, presenting a further challenge to studies of human vulnerability. As a consequence, all existing methods of estimating human vulnerability rely on elements of expert judgment and typically provide discrete vulnerability values for broad ranges of landslide process and intensity (AGS, 2007; Corominas et al., 2014; Lee & Jones, 2014; Safeland, 2011). Such ranges provided little power to accurately discriminate ex‐ante between trivial and life‐threatening events, as is necessary for risk assessment and mitigation.

While early risk analyses relied on heuristic vulnerability matrices, many modern analyses utilize the semiempirical estimates of human vulnerability from two place‐based studies (Corominas et al., 2014; Lee & Jones, 2014). Finlay (1996) compiled a data set of 27 fatal or near‐fatal landslides, primarily in Hong Kong. Single‐valued estimates of human vulnerability were provided in an event tree, associated with scenarios of landslide debris striking a building only, intruding into a building, or causing complete structural collapse. The data set does not include the number of people exposed to each landslide, and thus, the probability of death or injury of an exposed individual cannot be directly estimated. Michael‐Leiba et al. (2005) computed human vulnerability as the ratio of fatalities to building occupants in 24 landslides in the Australian landslide database, three of which involved fatalities. Based on these events, they produced an average human vulnerability of 0.1 for all landslides and recommended heuristically reducing this value to 0.05 to account for intrinsic underrepresentation of nonfatal landslides in the database (Michael‐Leiba et al., 2005). Most modern landslide risk assessments utilize the findings of these pioneering studies; however, the results may not be particularly transferable, as they are constrained by the limited types of landslides, process intensities, building stock, and socio‐behavioral idiosyncrasies of the original study locations (AGS, 2007; Lee & Jones, 2014). Furthermore, vulnerability estimates such as these only inform the potential for loss rather than strategies to reduce such losses (Papathoma‐Kohle et al., 2015).

### 2.4 Landslide Mortality

Mortality refers to *the rate of death within a population*. Here we adopt the term, using landslide mortality to refer not only to the rate of death in landslide incidents but also more generally to the contributing human factors and actions. Although landslide vulnerability is widely understudied, the underlying causes of landslide mortality have rarely even been discussed in academic literature (Kennedy et al., 2015). A handful of studies describe the patterns of injury and death in landslides (Gueri & Perez, 1986; Homma et al., 2016; Langdon et al., 2019; Memchoubi et al., 2012; Pereira et al., 2013; Sheeju et al., 2019). However, we are aware of only two studies that analyze the situations and behaviors that contribute to these outcomes (Agrawal et al., 2013; Sanchez et al., 2009). In practical terms, this represents a critical gap in our understanding of the human consequences of landslides, leaving decision‐makers, first responders, and at‐risk citizens with little scientific guidance for reducing human losses.

### 2.5 The Role of Human Behavior in Landslide Mortality

Ex‐post evidence shows that landslide damage to structures scales with the process intensity, measured as flow velocity, impact pressure, inundation depth, or failure volume (Fuchs, Keiler, et al., 2019). While the vulnerability of structures and the vulnerability of the people inside them are conceptually linked (Du et al., 2013; Finlay, 1996), the relationship between the two is unclear, and they may be dramatically different in similar processes (Fell, 1994). Some researchers have suggested there is a process intensity threshold at which the probability of death of indoor populations dramatically increases due to common structural design elements. Debris intrusion through windows is credited with the exponential increase in building damage at debris heights of 1.0 – 1.7 m (Papathoma‐Kohle, 2012, 2016; Totschnig et al., 2011), and Massey et al. (2019) noted that fatalities become more likely when inundation depths exceed the bottom window height (typically 1.0–1.4 m), evinced by three fatal landslides in New Zealand.

Finlay (1996) notes that indoor populations have a high vulnerability (>0.8) if they are buried by intruding debris or if the building collapses. Functional destruction of masonry structures is estimated to occur at debris heights greater than 2.5 m (Akbas et al., 2009; Fuchs, Keiler, et al., 2019; Quan Luna et al., 2011), while the lateral load‐bearing capacity of a typical timber‐frame wall is exceeded by debris heights of 1.4–1.6 m (Massey et al., 2019).

Based on structural vulnerability, we would expect mortality to approach 100% once the structural resistance is exceeded. However, there are numerous cases of humans surviving large landslides even when inundation depths exceed 1–2 m (e.g., Diver, 1999; Metivier‐Hart, 2017). A direct relationship between structural and human vulnerability ignores the inherent differences in coping potential between individuals and the ability of humans to act to dynamically change their vulnerability (Crozier & Glade, 2005). In this work, we test the idea that human behavior—rather than process intensity—governs landslide mortality over a range of process intensities.

## 3 Methods

### 3.1 Construction of the Fatality Data Set

We reviewed academic literature, government reports, news stories, survivor accounts, coroner reports, and legal filings to compile a data set of landslide events that impacted occupied structures. The basic information required for inclusion in the data set was the flow depth of debris at the structure, the number of occupants, and the outcome for the exposed individuals (survived or deceased). We recorded basic information about the landslide (date, time of day, and mode of failure), structure (construction material and location), and individuals (age and gender). When available, we also used first‐ or second‐hand accounts to reconstruct individuals' prior knowledge of the threat, location in the structure, and behavior around the time of the impact, using proxies for deceased individuals (Sanchez et al., 2009). For deceased victims, we compiled the cause of death when coroner reports were available. For survivors, we recorded if they escaped on their own or were rescued by others and, if rescued, the time of rescue and relation to the rescuer. We were unable to reconstruct complete information in all fields for all individuals. No direct human subjects research was performed as part of this work, and all sources are public record. Identifying information, even when publicly available, was anonymized to protect individuals' privacy.

The influence of landslide inundation depth on the probability of death was examined through nonlinear regression. Binary logistic regression was used to assess the impact of demographic, situational, and behavioral factors on human mortality. The inclusion criteria introduce a nonrandom sampling bias, although it is not systematic for most variables. News reports that include inundation depth are infrequent unless the event is particularly noteworthy, such as cases of partial burial or dramatic rescues, or contains other human‐interest elements. Thus, we anticipate the underrepresentation of extreme low‐ and high‐intensity events associated with total survival and total mortality, respectively. We do not believe the reporting bias systematically affects the results other than understating the fit of statistically derived vulnerability curves at the extremes.

### 3.2 Uncertainties Related to Postevent Reconstruction

Reconstructing technical details of rapid, traumatic events, sometimes many years after they occurred, has many sources of uncertainty. We assigned qualitative levels of confidence (low, medium, and high) to flow depth estimates based on the method through which they were produced.

Without photographic evidence during a landslide, flow depths are most reliably estimated from measuring mudlines. Such data are highly ephemeral and rarely collected in the chaos immediately following a large‐scale disaster (Kean et al., 2019a). In lieu of such data, postevent debris deposition depth can be used as a proxy for the flow depth (Akbas et al., 2009; Fuchs, Heiser, et al., 2019). Although also altered by erosion, rescue operations, or recovery and rebuilding, landslide deposits may last months to years after an event and be measured in ground surveys or elevation differencing (e.g., Wartman et al., 2016). Where either of these methods was used, we categorized flow depth measurements as high quality. Estimates of flow depths based on witness recollections, especially when they were gauged using objects of known height, were regarded as medium quality. We categorized as low quality the minority of cases in which flow depth was approximated nonnumerically, for example, “chest high” or “waist high.” In such cases, we must ask, “whose chest?” We used average biological measurements for the country of origin and gender of the victim to estimate the likely range of flow heights. This included cases in which victims were reported as being buried up to their chest (waist, neck, etc.), with the assumption that such approximations are only relevant to a standing (rather than prone) human. When referring to the landslide process intensity, we use the generic term “inundation depth.”

Issues of scale limit the back‐analysis of past events. At the scale of a community, a single flow depth may be associated with an entire structure (e.g., Kean et al., 2019b). However, at the scale of a building, it is unlikely that all rooms will be inundated equally (Quan Luna et al., 2011). The disparity between the scale of reported flow depths (often for an entire structure) and the scale of exposed individuals (who may be anywhere within the structure, including on upper floors) adds further uncertainty to postevent analysis (Totschnig & Fuchs, 2013).

### 3.3 Vulnerability Curves

Landslide vulnerability curves mathematically link relative damage from none (zero) to complete (one) to the process intensity for each element at risk (Ciurean et al., 2017). The production of robust landslide vulnerability curves is data intensive, requiring extensive data sets of damage‐intensity pairs for individual elements at risk (e.g., per building), making vulnerability curves rare and typically tied to a specific event and region (Papathoma‐Kohle et al., 2015; Totschnig & Fuchs, 2013). Many proxies for process intensity have been used in vulnerability curves for physical infrastructure, such as landslide area (Galli & Guzzetti, 2007), momentum flux (Prieto et al., 2018), impact pressure (Zhang et al., 2018), velocity (Kang & Kim, 2016), volume (Winter et al., 2013), and inundation depth (Quan Luna et al., 2011; Totschnig & Fuchs, 2013). We adopted inundation depth, as it is one of the most easily reconstructable landslide characteristics ex‐post‐facto and is relevant to a primary cause of death in landslides (suffocation due to burial).

In landslide risk analysis, human vulnerability may be expressed as the probability that an individual will be killed in a landslide (Corominas et al., 2014). The probability of death for an individual in a given structure can be calculated as the ratio of fatalities to occupants (Michael‐Leiba et al., 2005):

$$P_{D}=\frac{\text{number of fatalities}}{\text{number of occupants}}.$$

The basic mathematical requirements for a vulnerability curve are that it (1) must define vulnerability within the confined interval [0, 1], passing through the origin, and (2) be monotonically increasing within the interval [0, +∞) of the independent variable (Papathoma‐Kohle et al., 2012). In light of these criteria, we chose a modified Weibull cumulative distribution as the underlying model to represent human vulnerability to landslides, of the form

$$V=1-e^{-a*I^{b}},$$

where *I* is the landslide intensity measured as inundation depth (Totschnig et al., 2011).

## 4 Data Set

### 4.1 General Statistics

Our data set consists of 334 exposed individuals in 95 impacted buildings and 38 unique landslide events between 1887 and 2019. The gender distribution of exposed individuals is 44% male and 36% female, with the remainder unknown. Victim ages range from 4 months to 91 years with a mean of 36.4 ± 24.2 years. Inundation depths range from 0.2–11.3 m (8 in. to 37 ft) with a mean of 2.8 ± 2.3 m. Fifty‐seven percent of the inundation depths were deemed of “high” quality, 28% of “medium” quality, and 15% of “low” quality, reflecting the high proportion of cases which came from two well‐studied landslide events in Washington and California. These two events, the 2014 SR 530 “Oso,” Washington, flowslide, and the 2018 Montecito, California, debris flows, contribute 39% of the individuals and 52% of the structures in our data set. Forty‐nine percent of all exposed individuals were killed, while 20% were rescued by others, and 28% escaped or self‐rescued. In the remaining cases, all we know is that the individual survived.

### 4.2 Landslide Location

Due to the specific inclusion criteria, the data set is not representative of global landslide‐human interactions and is subject to reporting bias based on the existence of English language media accounts and academic studies. Most of our data come from the United States, with 36% of total landslides events, 73% of impacted structures, and 75% of affected individuals, reflecting the limitations of an English language search and the intense scrutiny of fatal landslides in the United States (Kirschbaum et al., 2010). Reporting bias is especially pronounced when considering countries by the United Nations threshold of economic development (United Nations, 2020). Economically developing countries represent 42% of events, 17% of structures, and 15% of individuals, although global data sets indicate that these nations experience greater numbers of fatal landslides and overall fatalities than economically developed countries (Dowling & Santi, 2014; Froude & Petley, 2018; Kirschbaum et al., 2010; UNISDR, 2009).

### 4.3 Cause of Death

Of the 157 decedents, the primary cause of death is known for 77 (49%). Traumatic injury was the immediate cause of death in 66 cases, while 8 individuals died by mechanical asphyxiation and 3 by drowning. Traumatic injury is a common cause of death in other landslides (Table 1). In a single landslide event in July 2011 in India, blunt force trauma to the head and vital organs was the cause of death in five out of the six fatalities (Memchoubi et al., 2012). In a 2019 landslide in Kerala, India, blunt force trauma was the primary cause of death in 18 out of 19 fatalities (Sheeju et al., 2019). A 2013 landslide in Oshima, Japan, resulted in a high proportion of nonfatal, severe chest and pelvic trauma, which the authors extrapolated as the presumed cause of death among decedents, although no postdeath analysis was performed (Homma et al., 2016).

**Table 1:** Primary Cause of Death in Selected Landslide Disasters (Percent)

Location	Mechanical asphyxia	Simple asphyxia	Traumatic injury	Reference
India (2011)	—	1 (17)	5 (83)	Memchoubi et al. (2012)
India (2019)	—	1 (5)	18 (95)^a^	Sheeju et al. (2019)
Micronesia (2002)	39 (91)	—	4 (9)	Sanchez et al. (2009)
Brazil (2011)	(75)	(25)	—	Pereira et al. (2013)
California (2018)	—	—	17 (100)	SBCSO (2018)
Washington (2014)	—	—	43 (100)	Snohomish County MEO (2014)
Australia (1995)	8 (44)	1 (6)	9 (50)^b^	Hand (2000)
Australia (2011)	—	2 (100)	—	Barnes (2012)
New Zealand (1998–2013)	3 (60)^c^	—	2 (40)	Massey et al. (2019)

^a^ Asphyxia was a contributing cause in 10 cases.

^b^ Asphyxia was a contributing cause in two cases.

^c^ Type of asphyxia not specified in two cases.

In contrast, a study of landslide fatalities after a series of storm‐triggered landslides in Chuuk, Micronesia, in 2002 found that suffocation by burial was the primary cause of death in approximately 90% of decedents, regardless of their location inside or outside of a home (Sanchez et al., 2009). In January 2011, a tropical storm triggered flooding and mudslides around Rio de Janeiro, Brazil, killing 845. The primary causes of death were mechanical and simple asphyxia, respectively. One third of the deceased experienced a traumatic injury, but it was not determined to be the primary cause of death in any case (Pereira et al., 2013). Out of five landslide fatalities in New Zealand 1998–2013, asphyxia (unspecified) was the primary cause of death in three cases (Massey et al., 2019). In one of these cases, the debris flow entered the dwelling but did not cause its complete collapse (Beetham, 2012).

The nature of fatal and nonfatal injuries appears to be a function of the landslide mode and the location of the victim. Both the 2014 Washington and 2018 California events were highly fluid but contained large quantities of boulders, trees, and massive debris, such as cars and disintegrated structures (Kean et al., 2019a; Keaton et al., 2014). Most of the decedents in these events were indoors at the time of inundation, and their homes were partially or entirely destroyed. This may have temporarily protected the victims from burial and suffocation but also increased their chances of receiving a fatal traumatic injury from moving furniture or structural elements.

While the 2002 Micronesia and 2011 Brazil events were also characterized by highly fluidized landslides, the construction and contents of residences in these areas likely contributed to the lower percentage of deaths caused by traumatic injury. Sanchez et al. (2009) found that of 22 indoor decedents, 68% sheltered in structures with concrete walls, which are less likely to collapse in small or midsized debris flows.

## 5 Results

### 5.1 Probability of Death and Critical Depth

The likelihood of dying in a landslide generally increases with increasing process intensity. The regression statistics indicate a mild correlation between human vulnerability and inundation depth. The probability of death rapidly increases between 0 and 2 m, although no fatalities are recorded below inundation depths of 0.8 m. Based on experience in New Zealand and Europe, we believe this is due to the intrusion of debris into buildings through structural weak points such as windows, commonly set at heights of ~1 m, after which debris may overwhelm and bury occupants (Massey et al., 2019; Totschnig et al., 2011).

**Figure 1:**
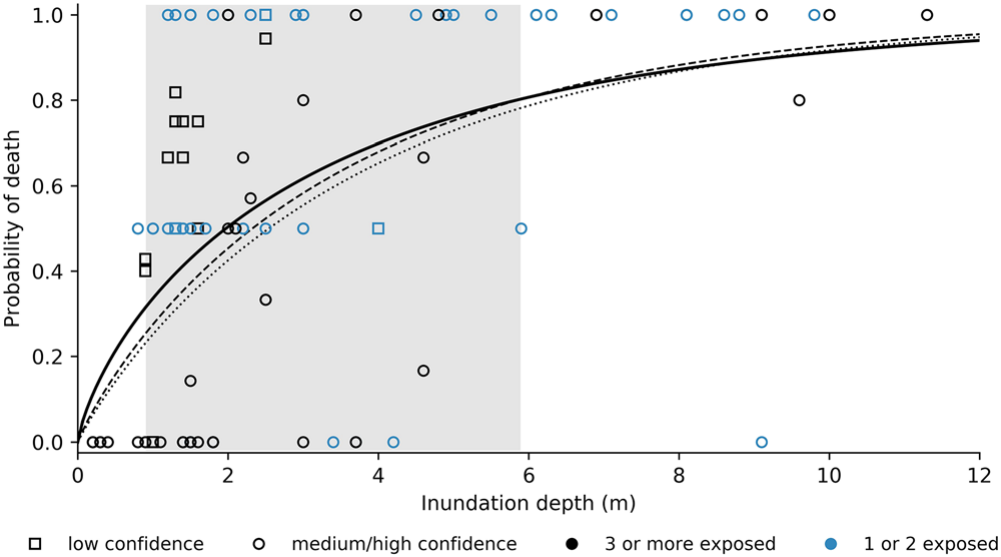
Human vulnerability curve based on the entire data set (solid line). Considering only cases in which more than two people are exposed (dotted line) or only medium/high confidence cases (dashed line) has little effect on the relationship. One additional total fatality case (30 m inundation depth) is not shown but included in all regressions. Between inundation depths of 0.9–5.9 m (gray shading), there is no statistical relationship between the probability of death and process intensity, suggesting that there is a critical zone in which other factors dominate landslide mortality.

Between inundation depths of 0.9–5.9 m, the mortality rate varies widely, encompassing 82% of exposed individuals and 76% of structures. Only one fatality occurred at depths shallower than 0.9 m, while the maximum inundation depth survived by an individual is 9.6 m. Approximately 90% of individuals were in a structure impacted by less than 6 m of debris, including 99% of survivors. Within the zone of 0.9–5.9 m, the probability of death has no correlation with inundation depth through nonlinear regression or binary logistic regression (depth binned in 1 m increments), suggesting that this is a critical zone for human mortality (Figure 1). While the exact values of 0.9 and 5.9 are somewhat arbitrary, they fit the observations and rationale of prior literature as well as the range of inundation depths compiled for this work in which both survivors and decedents were observed.

Fifty‐three percent of structures only had one to two occupants, reflecting the prevalence of adults living singly or with a partner. Since the probability of death is a function of the number of people exposed, values of 0, 0.5, and 1 are over represented in our data set. This led to additional uncertainty, particularly at the extreme ends of the range of inundation depths. For instance, in the case of the second‐highest inundation depth that was survived (9.1 m), the victim was the only person home, resulting in a probability of death of zero. However, the victim survived with severe injuries in very favorable circumstances, while all four occupants of an adjacent home were killed, indicating that the actual probability of death in this home was closer to one. Considering only buildings in which three or more people are exposed mildly improves the fit of the vulnerability function (Table 2) but only minimally alters the form of the final vulnerability function (Figure 1.)

**Table 2:** Regression Parameters for the Vulnerability Functions Shown in Figures 1, 2, 3

Subset	*a*	*b*	Pseudo *R* ^2^	*RMSE*	*n*
More than two individuals exposed	0.2899	0.9343	0.415	0.302	46
Inundation depths 0.9–5.9 m	0.4917	0.5164	0.053	0.376	72
Medium/high confidence cases	0.3216	0.9115	0.320	0.354	82
Economically developing nations	0.8782	1.6095	0.480	0.139	17
Economically developed nations	0.2752	0.9520	0.344	0.341	79
**Final (all points)**	**0.4096**	**0.7758**	**0.277**	**0.348**	**96**

We also tested the influence of cases in which the debris depth was estimated with low confidence. The regression results indicated a slightly weaker fit when using only the cases of medium or high confidence rather than using all cases (Table 2), indicating the data quality was insignificant relative to the other factors contributing to human vulnerability.

**Figure 2:**
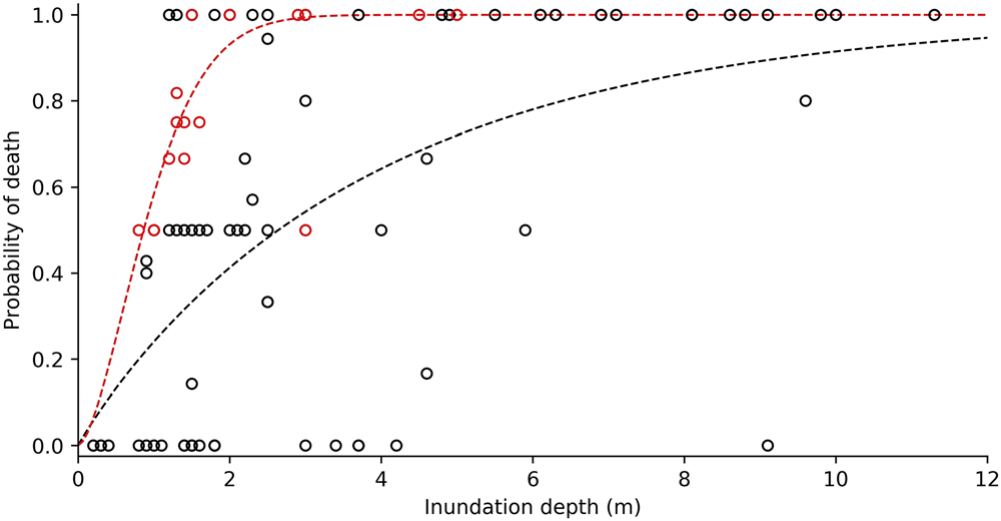
At inundation depths of approximately 2 m, predicted human vulnerability in economically developing nations (red dashes) is over 50% higher than in economically developed nations (black dashes). One additional total fatality case (30 m inundation depth) is not shown but included in the regression for developing nations.

Disaggregating by the economic development of the host nation suggests a strong socioeconomic component to human vulnerability (Figure 2). Human vulnerability in economically developing nations shows less scatter than in developed nations (Table 2), although due to the low number of data points (17) and the lack of zero‐fatality cases, there is potential for overfitting. Even so, economically developing nations have significantly greater human vulnerability than developed nations at almost all inundation depths. Although vulnerability curves for both economically developing and developed nations show the highest increase in vulnerability in the 0–2 m range, the increase is almost twice as rapid for developing nations. In developing nations, the probability of death reaches ~100% at 2.5 m, while the corresponding probability in developed nations is only 50%. Considering only cases of inundation depth 0.9–5.9 m, individuals in developing nations had six times the likelihood of death of those in developed nations (*p* value < 0.001).

**Figure 3:**
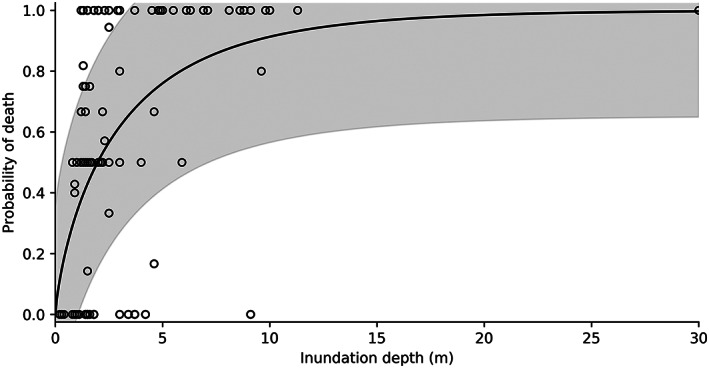
Human vulnerability to landslides over the range of common landslide inundation depths (*n* = 96). Gray shading represents ± one standard deviation.

Figure 3 shows the final vulnerability curve for the entire range of inundation depths represented in the data set. Regression parameters are given in Table 2. The poor fit of the regression model indicates that caution must be used for any predictive application and highlights the need for more human vulnerability data to disentangle the influence of additional complicating factors. Nevertheless, the explicit data scatter has the advantage of being statistically quantifiable for use in probabilistic analyses, as has been done for vulnerability curves for other types elements at risk (Massey et al., 2019), as well as in other aspects of landslide risk assessment (McDougall et al., 2012; Schilling, 2014).

### 5.2 Rescue and Time to Rescue

The first minutes after a landslide are critical. Of victims who were rescued, 77% were first located by neighbors and 11% by emergency response personnel. Due to their proximity, neighbors located and began to rescue landslide victims more rapidly than emergency personnel who often had to travel miles to reach the landslide site. Of those located by neighbors, the majority (68%) were found in the first minutes after the event, with only five victims (10%) being located more than an hour after the event. Although the time at which emergency personnel located and began the rescue of landslide victims is known in only four cases, the minimum time is 1 hr with a maximum time of 12 hr. Rescues begun by neighbors were often completed by emergency personnel, as specialized equipment was needed to extract buried victims.

### 5.3 Demographic and Situational Risk Factors

#### 5.3.1 Gender and Age

Our data show an overrepresentation of men, with a male to female ratio of 1.24 compared to 1.02 globally and 0.98 in the United States, from which the majority of our data comes (United Nations, 2019). The reasons for this are unclear. Since almost all cases are from residential structures, occupational or recreational overexposures to hazard are unlikely causes (HR Wallingford, 2006), although the difference may be explained by the relatively high proportion of individuals for which gender is not known (19%). Women are slightly more likely to be killed than men, although the correlation is not statistically significant (Table 3).

**Table 3:** Crude Odds Ratio (OR) for Factors Potentially Associated With Mortality in Landslides of Inundation Depths 0.9–5.9 m

Factor	Deaths	Survival rate (%)	OR	95% CI	*p* value
Economic development					
developed	92	59.6	ref		
developing	37	19.6	6.077	2.800–13.192	**< 0.001**
Gender					
female	46	49.5	1.356	0.785–2.342	0.274
male	52	57.0	ref		
Age					
0–19 (children)	24	57.1	ref		
20–39	22	37.1	2.256	0.949–5.365	0.066
40–59	22	29.0	3.259	1.275–8.335	**0.014**
60–79	13	38.1	2.167	0.775–6.054	0.140
80–99 (elderly)	3	57.1	1.000	0.204–4.893	1.000
Construction material					
rigid	23	37.8	3.119	1.495–6.506	**0.002**
flexible	59	65.5	ref		
Distance from slope (m)					
0–99	53	50.0	1.622	0.927–2.837	0.090
100–499	6	0	n/a	n/a	n/a
500–999	10	54.5	1.351	0.531–3.438	0.527
>1,000	37	61.9	ref		
Time of day					
12–8 p.m.	7	53.3	1.157	0.379–3.534	0.798
8 p.m. to 4 a.m.	84	53.1	1.169	0.674–2.029	0.578
4 a.m. to 12 p.m.	31	56.9	ref		
Awareness					
not aware	32	36.0	8.081	3.858–16.924	**< 0.001**
aware	22	82.0	ref		
Protective action					
no	39	51.9	5.014	2.484–10.123	**< 0.001**
yes	15	84.4	ref		
Floor occupied					
ground	77	60.1	11.948	1.563–91.356	**0.017**
upper	1	94.7	ref		

*Note*. *p* values associated with statistical significance (<0.05) are in bold.

Individuals between 40 and 59 years of age were 3.25 times more likely to be killed in a landslide than those from 0–19 years (Table 3). Children (0–19 years) and the elderly (80–99 years) have the highest survival rates of all age groups, both 57%. We suspect sampling bias is partially responsible for the high relative survival rate among children and the elderly. Children's ages were almost always included in news reports, whereas midrange adult ages were often only available from obituaries. Additionally, among the 115 individuals for which we could identify the occupied floor at the time of the landslide, 82% of individuals on the second or third floors were under the age of 40. Eleven percent of children were on upper floors, a far safer location in a landslide impacted structure, as is discussed in section 5.4.2.

#### 5.3.2 Construction Material

The physical vulnerability of structures, and thus also their occupants, is a function of the construction material, with timber‐frame structures having less resistance to landslides and rock falls than masonry or concrete structures (Massey et al., 2019). We differentiate between rigid construction materials, including masonry, stone, and reinforced concrete, and more flexible materials such as wood, bamboo, and plastic. The latter category dominates the data set at 76% of the structures, while rigid materials were used in only 9% of structures. Surprisingly, individuals in rigid structures were 3.1 times more likely to be killed than those in the flexible structures (Table 3). Considering only those structures impacted by 0.9–5.9 m of debris, the median inundation depth was 2.7 m for rigid structures and 1.8 m for flexible structures, suggesting that the overrepresentation of large events impacting rigid structures helps to explain this difference. The structural resilience of rigid materials may be both a blessing and a curse. Although debris may exert less damage on rigid structures at low and moderate inundation depths, if it intrudes into the building through structurally weak areas such as windows and doors, debris may fill the building rather than exiting, thus trapping and burying the occupants. At inundation depths capable of collapsing buildings regardless of construction material, falling masonry or concrete slabs may be more deadly than the landslide itself (e.g., Hand, 2000; Hudson, 1982).

#### 5.3.3 Distance From Slope

While the exact duration of warning prior to inundation is not known in most cases, the distance of an impacted structure to the slope where the landslide initiated can serve as a proxy for time. The odds of death increase with decreasing distance, with individuals less than 100 m from the slope 1.6 times more likely to be killed, although the results are not statistically significant (Table 3). We suspect that this is due to decreased time to identify and react to approaching landslide debris, although the people closest to the slope may also be more likely to notice warning signs of an impending landslide such trees cracking, sudden changes in surficial water flow, or outrunner rocks and soil. No statistically significant relationship exists between awareness and distance from slope for individuals in our data set.

#### 5.3.4 Time of Day

Time of day has no clear influence on landslide mortality (Table 3). However, individuals were 15–20 times more likely to be aware of an imminent threat in the morning (4 a.m. to 12 p.m.) than in the night (8 p.m. to 4 a.m.) or evening (12–8 p.m.; *p* values < 0.05). Twice as many people were killed during the night than during the rest of the day, reflecting the high proportion of people in their homes at this time. People killed by landslides while outside—more likely during daytime hours—are not included in our results.

### 5.4 Behavioral Risk Factors

#### 5.4.1 Awareness and Mitigative Action

Based on firsthand accounts of landslide survivors, we were able to coarsely identify their awareness of an imminent threat and their subsequent actions. This information cannot be precisely known for those who were killed, so we relied on the survivor accounts as proxies for the deceased. We were not able to reconstruct the extent of forewarning; in some cases, it may have been as long as tens of minutes. However, in all cases, the individuals exposed to landslide inundation did not have sufficient time, will, or means to evacuate.

Awareness of an approaching threat, even if its location and nature were unknown, sharply decreased the odds of death, with those who were not aware eight times more likely to be killed (*p* value < 0.001; Table 3). Three quarters of individuals who recognized a threat took some form of protective action, such as moving away from the perceived direction of the threat, escaping vertically to a higher floor or the top of furniture, or sheltering in a prepared refuge area in their home. However, a significant minority of individuals either took no action or moved closer to the oncoming landslide out of curiosity. Those who took no protective action were five times more likely to be killed (*p* value < 0.001). The survival rate among those who were aware of a threat was 82%, while among those who took protective action, it increased to 84%.

#### 5.4.2 Floor Occupied

The exact location of individuals in a home was rarely available, but in 77% of cases we could identify the floor occupied at the time of the landslide. The survival rate of individuals on the second or third floors, including attics and roofs, was 95%, with those on the ground floor 12 times more likely to be killed (*p* value < 0.001; Table 3). The only known case of a second floor fatality involved the violent destruction of the entire home.

### 5.5 Key Actions

In the 1970s, health professionals pioneered the concept of “positive deviance,” or the uncommon, beneficial practices of a few members of an at‐risk community that lead to better outcomes than those of their neighbors (Wishik & Van der Vynckt, 1976; Wray, 1972). Such practices are typically affordable, acceptable, and sustainable in a community because they are already practiced by at‐risk peers (Marsh & Schroeder, 2002). The concept of positive deviance can be adopted for disaster risk reduction. Engineering, political, or societal solutions to reduce risk may be unaffordable, unpalatable, or infeasible; however, behavioral change is a potent means for individuals to reduce their own risk. As we examined survivor stories, we identified six key, “positively deviant” actions that lead to beneficial outcomes.

Before a landslide event:

1. *Be informed about potential hazards and talk to people who have experienced them*: Prior experience with natural hazards is associated with greater preparation, more realistic perception of risk to future hazards, and enhanced ability to cope during hazardous events (Becker et al., 2017; Dunn et al., 2016; Hoffmann & Muttarak, 2017; Sattler et al., 2000). In two cases, individuals with prior firsthand experience of landslides recognized the signs of oncoming debris before seeing it and dashed to safer areas, narrowly escaping death.
2. *Move areas of high occupancy, such as bedrooms, upstairs, or to the downhill side of a home*: Bedrooms are often on the uphill side of residential dwellings, placing occupants closest to potential landslide hazards during the night, when they are least likely to be aware of an imminent threat (Taig et al., 2015). One family survived a debris flow by sheltering in a downhill bedroom, while the two uphill bedrooms were completely inundated (McPhee, 1989). If moving bedrooms is unfeasible, moving beds away from exterior walls may also reduce risk (Faber, 2016).

During a landslide event:

- *Escape vertically*: Mortality rate dramatically decreases for those above the ground floor of a landslide‐impacted structure, even when the entire home is destroyed. Two survivors of the 2014 Oso landslide in Washington State credited this with saving their lives: “Being upstairs [in our home], I think that gave us a chance” (Keaton et al., 2014). For those in one‐story homes without roof access, moving higher onto countertops and furniture protected them from suffocation or being swept away (Cobery et al., 2012; McPhee, 1989).
- *Identify and relocate to interior, unfurnished areas*: Areas such as closets, bathrooms, and interior hallways can offer additional protection in landslides disasters. These small spaces are less likely to collapse due to the density of structural elements and are generally free from unsecured furniture, which could pin or crush a person. In an exceptional case of survival, a victim of the 2005 La Conchita, California, landslide dove into a closet that had been prepared as a refuge area and survived being buried by over 9 m of debris (Metivier‐Hart, 2017).
- *Open downhill doors and windows*: Doors commonly open inward, making it easier for landslide debris to enter structures than exit. In the case of fluid landslides, this may lead to a buildup of debris than can bury and suffocate occupants or, in extreme cases, develop enough pressure to rip apart the structure. We were surprised by the number of quick‐thinking individuals who opened downhill doors or kicked out windows to allow debris to flow through their home. However, we also note that individuals who did so after debris had begun to accumulate risked being swept out of their homes (McPhee, 1989).

If caught in landslide debris:

- *Continue to make noise and motion*: It is rare for a landslide to engulf an entire community, meaning that the family and neighbors of victims usually begin rescue activities within minutes after the event. In most cases, victims are found quickly, even if they cannot be fully rescued without professional aid. Buried survivors who were successfully rescued often made noise through calling, whistling, or tapping on debris. Those partially buried attracted rescuers through waiving, and in at least one case, a fully buried man was able to poke a stick to the surface and use it to flag down rescuers.

Conversely, we also identified key actions which put individuals at greater risk:

1. *Opening a door out of curiosity*: It is a typical human response to move toward unknown or unfamiliar phenomena, whether to identify a potential threat or out of curiosity (HR Wallingford, 2006). In at least four cases, as a result of hearing unfamiliar sounds or seeing mud flowing in the streets, residents opened their front door only to be swept away by a surge of debris.
2. *Sheltering behind or beside large furniture*: The high percentage of landslides deaths that occur due to blunt force trauma, even among victims who are indoors at the time, suggests that unsecured furniture is a significant contributor to landslide mortality. In the Crescenta Valley flood, a man was crushed by a piano propelled by debris (Cobery et al., 2012). When a debris flow in Los Angeles inundated the home of a family of four, the two teenage children almost drowned from being pinned between a bed and a wall by the force of the flow, while the parents, buoyed on the top of the bed, survived unscathed (McPhee, 1989).

## 6 Discussion

### 6.1 Physical Vulnerability

Human vulnerability to landslides increases with increasing process intensity, although the relationship is neither as clear nor as robust as for buildings (Fuchs, Keiler, et al., 2019). Differences in building construction and material quality, size, orientation, and flow characteristics introduce inherent uncertainty in structural vulnerability curves. Human vulnerability couples structural vulnerability, with its already significant degree of variability, with a diverse range of demographic, situational, and behavioral human factors that produce further scatter in the data. However, many human factors are not readily predictable ex‐ante for use in quantitative risk assessment. As such, human vulnerability curves such as Figure 3 provide a practical improvement on single‐valued, heuristic estimates by coupling vulnerability and process intensity while quantifying model uncertainty.

**Figure 4:**
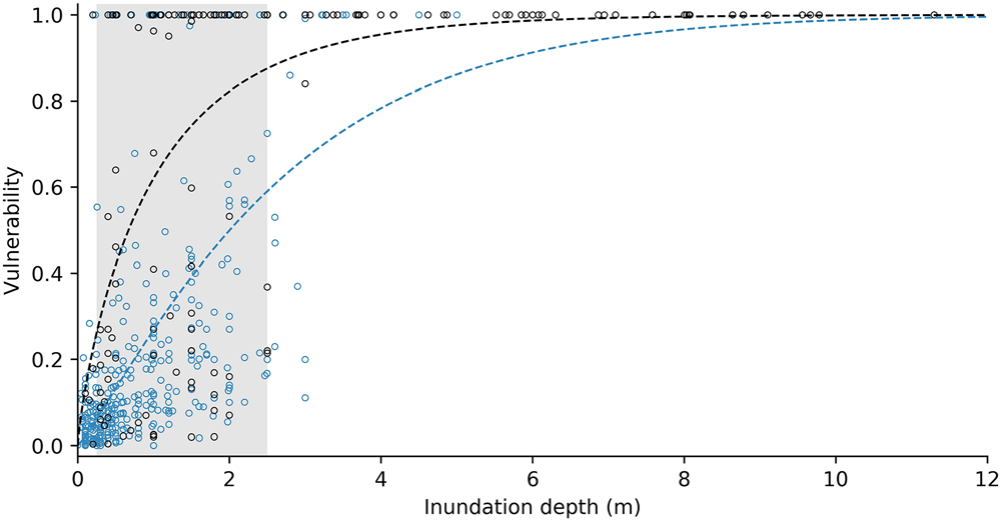
Compilation of published data sets of structural vulnerability to landslides. Structural vulnerability varies widely for inundation depths 0.25 – 2.5 m (gray shading), but above this range, almost all buildings are destroyed. Data from Pollock (2020).

Differences in the vulnerability curves for humans and buildings demonstrate the difficulty in mathematically linking structural and human vulnerability (Li et al., 2010; Uzielli et al., 2008). While the vulnerability of buildings is most varied between inundation depths of 0.25–2.5 (Figure 4), human vulnerability is most varied at greater inundation depths, between 0.9 and 5.9 m. The lower bound may be a function of anthropometric measures. Based on average leg to height ratios, Du et al. (2013) suggest a critical landslide depth of 0.8 m, although structures will shelter an indoor population until the debris intrudes through openings or exceeds the load capacity of a wall. Inundation depths of 2.5 m or greater result in the functional destruction, and often collapse, of almost all buildings regardless of construction material (Akbas et al., 2009; Tsao et al., 2010). However, 23% of the survivors in our data set occupied buildings inundated by at least this much debris, indicating that human vulnerability is not a simple function of either the vulnerability of the occupied structure or the process intensity; rather, within this zone, human behavior is the primary factor in landslide mortality.

Within the critical inundation depths of 0.9–5.9 m, the overall mortality rate is 47%. While this is lower than global landslide mortality rates produced by other methods (Kennedy et al., 2016), it is high relative to other environmental hazards, reflecting the violent nature of landslide processes (Alderman et al., 2012; Alexander & Magni, 2013; Dresser et al., 2016; Kennedy et al., 2015). While the language colloquially used regarding landslide victims such as “buried alive” (e.g., Metivier‐Hart, 2017) implies death by suffocation, traumatic injury is at least as common a cause of death. For indoor populations, unsecured furniture and collapsing structural elements may be more dangerous than the landslide itself, as is the case for earthquakes (Doocy et al., 2013; Glass et al., 1977).

### 6.2 Socioeconomic Pressures

Politically and economically disadvantaged populations suffer greater human losses in environmental disasters of all types, including landslides (Dowling & Santi, 2014; Kennedy et al., 2016; Santi et al., 2011). Our results suggest that a socioeconomic component to physical vulnerability is a dominant reason, with individuals in economically developing nations up to twice as vulnerable to landslides as those in developed nations. The rate at which landslide victims were rescued was lower in economically developing nations (15%) than in developed nations (20%), suggesting that fewer resources and personnel available for emergency response are contributing to greater landslide mortality. However, no individuals in developing nations were reported as self‐rescuing compared to 32% of landslide victims in developed nations, leading us to believe that there is a substantial reporting bias in the English language media, which omits landslide events that do not include fatalities or dramatic rescues.

### 6.3 Gender

Men are overrepresented in our data set, but this is likely a by‐product of uneven sampling rather than systemic overexposure to landslide hazards. Landslide fatality data sets from Italy, Switzerland, and Portugal show that in these countries far more men die in landslides than women, a difference attributed to increased occupational exposure and more risk‐taking behavior (Badoux et al., 2016; Pereira et al., 2016; Salvati et al., 2018; HR Wallingford, 2006). However, since most of the individuals in our data set were at home at the time of the landslide, the first explanation is unsatisfactory. In our data set, women had a slightly higher mortality rate than men, indicating that behavioral vulnerability associated with risk taking is also an insufficient explanation.

As in this work, Sanchez et al. (2009) found that women had a higher mortality rate than men during the 2002 Micronesia landslides, although it was not associated with a statistically significant increase in the probability of death. However, in the 2010 Uganda landslides, men were almost 2.5 times more likely to be killed (Agrawal et al., 2013), leading us to believe that the location of the victim plays an important role in the gender distribution of different landslide mortality data sets. In the Uganda event, more landslide injuries occurred outdoors, whereas in Micronesia more landslide fatalities occurred indoors, where the mortality rate between males and females tends to equalize (Pereira et al., 2016).

### 6.4 Age

Environmental disasters do not impact populations uniformly or at random (Hewitt, 1997; Wisner et al., 2004). A “saddle” shaped distribution of mortality rate by age, in which children and the elderly are disproportionately likely to suffer injury or death, has been observed in other natural hazards (Glass et al., 1977; Li et al., 2010; Zahran et al., 2008). Many explanations have been proposed, including that children and the elderly are less physically able to escape from hazardous areas or resist the physical trauma of a violent landslide or that children have a lesser understanding of the hazard and fewer life experiences to draw on for rapid decision‐making (HR Wallingford, 2006; Zahran et al., 2008). Indicator‐based frameworks for landslide vulnerability have adopted these results, assigning greater vulnerability to the young and aged (Eidsvig et al., 2014; Li et al., 2010; Park et al., 2016; Uzielli et al., 2008).

However, our results indicated that landslides might not “discriminate” by age in the same manner as other natural hazards. Middle‐age adults (40–59) were statistically more likely to be killed in a landslide than any other age group, contrary to the generally accepted view of age‐based vulnerability. In part, this is because landslides are a rapid‐onset, localized hazard, which stratifies mortality by building, room, and floor, meaning that social norms that organize residential spaces by generation strongly control who will be injured or killed in a landslide. In U.S. homes, master bedrooms—more likely occupied by a middle‐aged adult(s) than by children or elderly parents—are often located on the ground floor (Vollmer et al., 2005), where landslide impacts are the most severe. Since social norms and pressures vary by culture, region‐specific mortality‐age distributions may take different forms. In the 1995 Kobe, Japan, earthquake, individuals over 60 years old experienced higher mortality because many of the elderly lived in inexpensive and collapse‐prone wooden homes with heavy tiled roofs where, through custom and pragmatism, they slept on the ground floor (Kunii et al., 1995).

### 6.5 Construction Material

More durable construction material does not *necessarily* commute landslide risk. While buildings constructed out of masonry or concrete may be able to resist greater inundation depths than timber frame buildings prior to catastrophic failure, when they do collapse, it is often fatal for the occupants (Massey et al., 2019; Sanchez et al., 2009). Additionally, when masonry buildings collapse, they leave fewer cavities in which individuals can be sheltered (Coburn & Spence, 2002). Du et al. (2013) estimate that the vulnerability of humans during the total collapse of masonry and reinforced concrete structures is 1.9 and 1.3 times greater than for timber frame structures.

### 6.6 Hazard Awareness

Education and awareness of potential landslide threats are a common risk‐reduction strategy (Davis et al., 2020; Highland & Bobrowsky, 2008; WGS, 2017). In a study of residents' experiences during rock falls triggered by the 22 February 2011 earthquake in Port Hills, New Zealand, Taig et al. (2015) found that the ability to recognize approaching boulders and take evasive action likely saved the lives of multiple individuals. In particular, residents who have familiarized themselves with the local landslide history, the direction from which landslides will come, and landslide precursors such as extra surficial water, falling and bouncing rocks, ground tremors, and rumbling noises are more likely to take early and appropriate mitigative action such as evacuating, moving to the interior or downhill side of a home, or relocating to a higher floor, attic, or roof.

Furthermore, knowledge of the local landslide hazards increases the likelihood of an individual making advanced preparations, such as identifying or creating a structurally reinforced refuge space, relocating bedrooms to the downhill side of a home, and moving beds away from windows and doors (Faber, 2016; Metivier‐Hart, 2017; Taig et al., 2015). This indicates that outreach products that inform citizens of local landslide hazard and risk, landslide triggers and precursors, and protective actions during a landslide event are a critical component of landslide risk reduction (e.g., Davis et al., 2020; WGS, 2017; WGS & DOGAMI, 2015). However, public outreach and advisories must be unambiguous for their intended audience. In the days leading up to the 2018 Montecito debris flow disaster, different risk perceptions between scientists, emergency managers, and the public led to a false sense of security among residents of a designated “voluntary evacuation zone” (Hayden, 2018a). During the debris flow event, an ambiguous emergency SMS message instructing residents to “go to high ground” led some individuals to evacuate their homes only to be swept away (Hayden, 2018b).

### 6.7 Emergency Response

Few people simply walk away from being caught in a landslide. The significant percentage of survivors who were rescued suggests that an immediate emergency response may save up to 20% of potential fatalities. Such a reaction is often launched immediately by the surviving family and neighbors of the victims, as is the case in other environmental disasters (Clukey, 2010; Merchant et al., 2010). The presence of volunteer rescuers may increase the initial effectiveness of emergency response due to the rapidity in which they can locate trapped victims (Coburn & Spence, 2002). However, they may lack the specialized knowledge, training, and equipment necessary to perform triage, complete a rescue, and avoid becoming secondary victims themselves (Hand, 2000; Pereira et al., 2013; Zhang et al., 2015).

### 6.8 Landslide Morbidity

Finally, fatalities are not the whole story. Direct experience of a landslide or membership in a community affected by one—including first responders—may have repercussions that far outlast the emergency response and are not limited to those who were physically injured by the landslide. After the 1998 Sarno, Italy, debris flows, surviving community members reported financial, occupational, psychological, and domestic problems resulting from the event. Symptoms of posttraumatic stress disorder (PTSD) were almost universal among survivors (Catapano et al., 2001). Studies suggest that levels of PTSD, material damage, social conflict, and the likelihood of bereavement are higher among landslide victims than those of other natural hazards, while their loss of social support is greater (Norris et al., 2004; Yang et al., 2011). This may be due to the relatively high mortality rate in landslide disasters, as well as the complete material destruction, which often accompanies landslides, requiring survivors to relocate away from built‐in social support structures. Regardless of the reasons, landslides can have severe, persistent consequences that may not be apparent from immediate postdisaster assessments. The long‐term personal and community recovery necessary after a landslide disaster emphasizes the need for predisaster preparedness planning and postdisaster interventions that focus on more than only survival actions (Gowan et al., 2015).

## 7 Conclusions

At intermediate inundation depths, human behavior is the most significant factor in landslide mortality. Hazard preparation, situational awareness, and informed protective action such as moving to a higher floor or a prepared refuge space are potent and readily accessible means of lowering personal landslide risk. However, such strategies are predicated on scientific products such as landslide hazard and risk maps that are understandable, accessible, and communicable to the general public. The development of such products and education campaigns to put them in the hands of at‐risk populations are the first steps to landslide risk reduction. Rapid rescue operations after a landslide disaster save up to 20% of landslide victims but may be hindered by the long distances emergency personnel must travel to a disaster site. Thus, community programs to train citizen first responders could be a crucial step to saving lives. Finally, this work highlights the need for more human vulnerability data to be preserved in the aftermath of landslide disasters to inform landslide risk assessments, understand the complex situational and behavioral factors that contribute to landslide mortality, and design evidence‐based emergency‐response plans and outreach products to maximize survival rates in landslides.

## Conflict of Interest

The authors declare no conflicts of interest relevant to this study.

## Data Availability

The landslide fatality data set is available via the DesignSafe‐CI Data Depot at https://www.designsafe‐ci.org/data/browser/public/designsafe.storage.published//PRJ‐2866 (Pollock & Wartman, 2020). All data sources are public record. We have removed personally identifiable information to protect the privacy of individuals involved. No human subjects research was performed as part of this work.

## References

[gh2190-bib-0001] Agrawal, S. , Gopalakrishnan, T. , Gorokhovich, Y. , & Doocy, S. (2013). Prehospital and Disaster Medicine, 28(4), 314–321. 10.1017/S1049023X13000356 23746361

[gh2190-bib-0002] AGS (2007). Commentary on practice note guidelines for landslide risk management 2007. Australian Geomechanics, 42, 115–158.

[gh2190-bib-0003] Akbas, S. , Blahut, J. , & Sterlacchini, S. (2009). Critical assessment of existing physical vulnerability estimation approaches for debris flows. Strasbourg: Proceedings of Landslide Processes: From Geomorphologic Mapping to Dynamic Modelling.

[gh2190-bib-0004] Alderman, K. , Turner, L. , & Tong, S. (2012). Floods and human health: A systematic review. Environment International, 47, 37–47. 10.1016/j.envint.2012.06.003 22750033

[gh2190-bib-0005] Alexander, D. , & Magni, M. (2013). Mortality in the L'Aquila (Central Italy) earthquake of 6 April 2009. PLoS Currents, 5 10.1371/50585b8e6efd1 PMC3541886 23326762

[gh2190-bib-0006] Badoux, A. , Andrex, N. , Techel, F. , & Hegg, C. (2016). Natural hazard fatalities in Switzerland from 1946 to 2015. Natural Hazards and Earth System Sciences, 16(12), 2747–2768. 10.5194/nhess-16-2747-2016

[gh2190-bib-0007] Barbolini, M. , Cappabianca, F. , & Savi, F. (2004). Risk assessment in avalanche‐prone areas. Annals of Glaciology, 38, 115–122. 10.3189/172756404781815103

[gh2190-bib-0008] Barnes, M. (2012). Inquest into the deaths caused by the south‐east Queensland floods of January 2011. Brisbane: Office of the State Coroner.

[gh2190-bib-0009] Becker, J. , Paton, D. , Johnston, D. , Ronan, K. , & McClure, J. (2017). The role of prior experience in informing and motivating earthquake preparedness. International Journal of Disaster Risk Reduction, 22, 179–193. 10.1016/j.ijdrr.2017.03.006

[gh2190-bib-0010] Beetham, D. (2012). Ohope Beach landslide of 18 June 2011. GNS Science Report 2011/46. GNS Science.

[gh2190-bib-0011] Catapano, F. , Malafronte, R. , Lepre, F. , Cozzolino, P. , Arnone, R. , Lorenzo, E. , Tartaglia, G. , Starace, F. , Magliano, L. , & Maj, M. (2001). Psychological consequences of the 1998 landslide in Sarno, Italy: A community study. Acta Psychiatrica Scandinavica, 104(6), 438–442. 10.1034/j.1600-0447.2001.00512.x 11782236

[gh2190-bib-0012] Ciurean, R. , Hussin, H. , van Westen, C. , Jaboyedoff, M. , Nicolet, P. , Chen, L. , Frigerio, S. , & Glade, T. (2017). Multi‐scale debris flow vulnerability assessment and direct loss estimation of buildings in the Eastern Italian Alps. Natural Hazards, 85(2), 929–957. 10.1007/s11069-016-2612-6

[gh2190-bib-0013] Clukey, L. (2010). Transformative experiences for hurricanes Katrina and Rita disaster volunteers. Disasters, 34(3), 644–656. 10.1111/j.1467-7717.2010.01162.x 20298266

[gh2190-bib-0014] Cobery, A. , Lawler, M. , & Lawler, P. (2012). The Great Crescenta Valley Flood. Charleston, South Carolina: History Press.

[gh2190-bib-0015] Coburn, A. , & Spence, R. (2002). Earthquake protection. West Sussex: John Wiley & Sons 10.1002/0470855185

[gh2190-bib-0016] Corominas, J. , van Westen, C. , Frattini, P. , Cascini, L. , Malet, J. P. , Fotopoulou, S. , Catani, F. , van den Eeckhaut, M. , Mavrouli, O. , Agliardi, F. , Pitilakis, K. , Winter, M. G. , Pastor, M. , Ferlisi, S. , Tofani, V. , Hervás, J. , & Smith, J. T. (2014). Recommendations for the quantitative analysis of landslide risk. Bulletin of Engineering Geology and the Environment, 73, 209–263. 10.1007/s10064-013-0538-8

[gh2190-bib-0017] Crozier, M. , & Glade, T. (2005). Landslide hazard and risk: Issues, concepts and approach In Glade T., Anderson M., Crozier M. (Eds.), Landslide Hazard and Risk (Chap. 1, pp. 1–40). West Sussex: John Wiley & Sons 10.1002/9780470012659.ch1

[gh2190-bib-0018] Cruden, D. , & Varnes, D. (1996). Landslide types and processes In Turner K. & Schuster R. (Eds.), Landslides: Investigation and Mitigation (Chap. 3, pp. 36–75). Transportation Research Board: Washington.

[gh2190-bib-0019] Davis, L. , West, J. , Peek, L. , Hughes, K. , Joyce, J. , Schulz, W. , Godt, J. , Martinez, D. , Sanchez, G. , Perez, G. , Cardenas, C. , Hillebrandt, C. , Nieves, L. , & Hernandez, J. (2020). Landslide guide for residents of Puerto Rico. United States Geologic Survey Guidebook. https://www.usgs.gov/news/new‐landslide‐guidebook‐puerto‐rico‐residents

[gh2190-bib-0020] Diver, S. (1999). Survival. Sydney: Pan Macmillan Australia.

[gh2190-bib-0021] Doocy, S. , Cherewick, M. , & Kirsch, T. (2013). Mortality following the Haitian earthquake of 2010: A stratified cluster survey. Population Health Metrics, 11(1). 10.1186/1478-7954-11-5 PMC3648495 23618373

[gh2190-bib-0022] Dowling, C. , & Santi, P. (2014). Debris flows and their toll on human life: A global analysis of debris‐flow fatalities from 1950 to 2011. Natural Hazards, 71(1), 203–227. 10.1007/s11069-013-0907-4

[gh2190-bib-0023] Dresser, C. , Allison, J. , Broach, J. , Smith, M.‐E. , & Milsten, A. (2016). High‐amplitude Atlantic hurricanes produce disparate mortality in small, low‐income countries. Disaster Medicine and Public Health Preparedness, 10(6), 832–837. 10.1017/dmp.2016.62 27572097

[gh2190-bib-0024] Du, J. , Yin, K. , Nadim, F. , Lacasse, S. (2013). Quantitative vulnerability estimation for individual landslides. Paris: Proceedings of the 18^th^ International Conference on Soil Mechanics and Geotechnical Engineering.

[gh2190-bib-0025] Dunn, P. , Ahn, A. , Bostrom, A. , & Vidale, J. (2016). Perceptions of earthquake early warning on the U.S. West Coast. International Journal of Disaster Risk Reduction, 20, 112–122. 10.1016/j.ijdrr.2016.10.019

[gh2190-bib-0026] Eidsvig, U. , Papathoma‐Kohle, M. , Du, J. , Glade, T. , & Vangelsten, B. (2014). Quantification of model uncertainty in debris flow vulnerability assessment. Engineering Geology, 181, 15–26. 10.1016/j.enggeo.2014.08.006

[gh2190-bib-0027] Faber, E. (2016). Development of a landslide risk rating system for small‐scale landslides affecting settlements in Guatemala City. (*masters thesis*). Golden, Colorado: Colorado School of Mines.

[gh2190-bib-0028] Fell, R. (1994). Landslide risk assessment and acceptable risk. Canadian Geotechnical Journal, 31(2), 261–272. 10.1139/t94-031

[gh2190-bib-0029] Fell, R. , Ho, K. , Lacasse, S. , & Leroi, E. (2005). A framework for landslide risk assessment and management In Hungr O., Fell R., Couture R. & Eberhardt E. (Eds.), Landslide Risk Management (3–25). London: Taylor & Francis.

[gh2190-bib-0030] Finlay, P. (1996). The risk assessment of slopes. (*doctoral dissertation*). Sydney: University of New South Wales

[gh2190-bib-0031] Froude, M. , & Petley, D. (2018). Global fatal landslide occurrence from 2004 to 2016. Natural Hazards and Earth System Sciences, 18(8), 2161–2181. 10.5194/nhess-18-2161-2018

[gh2190-bib-0032] Fuchs, S. , Heiser, M. , Schlogl, M. , Zischg, A. , Papathoma‐Kohle, M. , & Keiler, M. (2019). Short communication: A model to predict flood loss in mountain areas. Environmental Modelling & Software, 117, 176–180. 10.1016/j.envsoft.2019.03.026

[gh2190-bib-0033] Fuchs, S. , Keiler, M. , Ortlepp, R. , Schinke, R. , & Papathoma‐Kohle, M. (2019). Recent advances in vulnerability assessment for the built environment exposed to torrential hazards: Challenges and the way forward. Journal of Hydrology, 575, 587–595. 10.1016/j.jhydrol.2019.05.067

[gh2190-bib-0034] Galli, M. , & Guzzetti, F. (2007). Landslide vulnerability criteria: A case study from Umbria, Central Italy. Environmental Management, 40(4), 649–665. 10.1007/s00267-006-0325-4 17638046

[gh2190-bib-0035] Glade, T. (2003). Vulnerability assessment in landslide risk analysis. Die Erde, 134, 123–146.

[gh2190-bib-0036] Glass, R. , Urrutia, J. , Sibony, S. , Smith, H. , Garcia, B. , & Rizzo, L. (1977). Earthquake injuries related to housing in a Guatemalan village. Science, 197(4304), 638–643. 10.1126/science.197.4304.638 17776257

[gh2190-bib-0037] Gowan, M. , Sloan, J. , & Kirk, R. (2015). Prepared for what? Addressing the disaster readiness gap beyond preparedness for survival. BMC Public Health, 15 10.1186/s12889-015-2440-8 PMC4647663 26576816

[gh2190-bib-0038] Gueri, M. , & Perez, L. (1986). Medical aspects of the “El Ruiz” avalanche disaster, Columbia. Disasters, 10(2), 150–157. 10.1111/j.1467-7717.1986.tb00580.x

[gh2190-bib-0039] Hand, D. (2000). Report of the inquest into the deaths arising from the Thredbo landslide. New South Wales: State Coroner.

[gh2190-bib-0040] Hayden, T. (2018a). “Internal records reveal mixed messages, missed opportunities before 1/9 debris flow.” Santa Barbara Independent. Available at: https://www.independent.com/2018/05/24/internal‐records‐reveal‐mixed‐messages‐missed‐opportunities‐before‐1‐9‐debris‐flow/. Accessed May 2020.

[gh2190-bib-0041] Hayden, T. (2018b). “Public survey exposes Montecito debris flow communication failures.” Santa Barbara Independent. Available at: https://www.independent.com/2018/06/14/public‐survey‐exposes‐montecito‐debris‐flow‐communication‐failures/. Accessed: May 2020.

[gh2190-bib-0042] Hewitt, K. (1997). Regions of risk: A geographical introduction to disasters. Essex: Addison Wesley Longman.

[gh2190-bib-0043] Highland, L. , & Bobrowsky, P. (2008). The landslide handbook—A guide to understanding landslides. *Circular 1325*. Reston, VA: U.S. Geological Survey.

[gh2190-bib-0044] Hoffmann, R. , & Muttarak, R. (2017). Learn from the past, prepare for the future: Impacts of education and experience on disaster preparedness in the Philippines and Thailand. World Development, 96, 32–51. 10.1016/j.worlddev.2017.02.016

[gh2190-bib-0045] Homma, Y. , Watari, T. , Baba, T. , Suzuki, M. , Shimizu, T. , Fujii, Y. , Takazawa, Y. , Maruyama, Y. , & Kaneko, K. (2016). Disaster Medicine and Public Health Preparedness, 10(2), 248–252. 10.1017/dmp.2015.167 26744090

[gh2190-bib-0103] HR Wallingford (2006). Flood risks to people. Defra/Environment Agency Flood and Coastal Defense R&D Programme FD2321/PR.

[gh2190-bib-0046] Hudson, R. (1982). Report on the rainstorm of August 1982. *GCO Report No. 7/82* . Hong Kong Geotechnical Engineering Office.

[gh2190-bib-0047] Jakob, M. , Stein, D. , & Ulmi, M. (2012). Vulnerability of buildings to debris flow impact. Natural Hazards, 60(2), 241–261. 10.1007/s11069-011-0007-2

[gh2190-bib-0116] Hungr, O. , Leroueil, S. , Picarelli, L. (2014). The Varnes classification of landslide types, an update. Landslides, 11(2), 167–194. 10.1007/s10346-013-0436-y

[gh2190-bib-0048] Kang, H.‐s. , & Kim, Y.‐t. (2016). The physical vulnerability of different types of building structure to debris flow events. Natural Hazards, 80(3), 1475–1493. 10.1007/s11069-015-2032-z

[gh2190-bib-0049] Kean, J. , Staley, D. , Lancaster, J. , Rengers, F. , Swanson, B. , Coe, J. , Hernandez, J. , Sigman, A. , Allstadt, K. , & Lindsay, D. (2019a). Inundation, flow dynamics, and damage in the 9 January 2018 Montecito debris‐flow event, California, USA: Opportunities and challenges for post‐wildfire risk assessment. Geosphere, 15(4), 1140–1163. 10.1130/GES02048.1

[gh2190-bib-0050] Kean, J. , Staley, D. , Lancaster, J. , Rengers, F. , Swanson, B. , Coe, J. , Hernandez, J. , Sigman, A. , Allstadt, K. , Lindsay, D. (2019b). Debris‐flow inundation and damage data from the 9 January 2018 Montecito debris‐flow event, U.S. Geological Survey data release, 10.5066/P9JQJU0E

[gh2190-bib-0051] Keaton, J. , Wartman, J. , Anderson, S. , Benoit, J. , dela Chapelle, J. , & Gilbert, R. (2014). The 22 March 2014 Oso Landslide, Snohomish County, Washington. Geotechnical Extreme Events Reconnaissance Report.

[gh2190-bib-0052] Kennedy, I. , Petley, D. , & Murray, V. (2016). Landslides In Koenig K. & Schultz C. (Eds.), Koenig and Schultz's disaster medicine: Comprehensive principles and practices (Chap. 42, pp. 716–723). Cambridge: Cambridge University Press 10.1017/CBO9781139629317.045

[gh2190-bib-0053] Kennedy, I. , Petley, D. , Williams, R. , & Murray, V. (2015). A systematic review of the health impacts of mass earth movements (landslides). PLoS Currents, 7 10.1371/currents.dis.1d49e84c8bbe678b0e70cf7fc35d0b77 PMC4423842 25969795

[gh2190-bib-0054] Kirschbaum, D. , Adler, R. , Hong, Y. , Hill, S. , & Lerner‐Lan, A. (2010). A global landslide catalog for hazard applications: Method, results, and limitations. Natural Hazards, 52, 561–575. 10.1007/s11069-009-9401-4

[gh2190-bib-0055] Kunii, O. , Akagi, M. , & Kita, E. (1995). The medical and public health response to the great Hanshin‐Awaji earthquake in Japan: A case study in disaster planning. Medicine Global Survival, 2, 214–226.

[gh2190-bib-0056] Langdon, S. , Johnson, A. , & Sharma, R. (2019). Debris flow syndrome: Injuries and outcomes after the Montecito debris flow. The American Surgeon, 85(10), 1094–1098. 10.1177/000313481908501004 31657301

[gh2190-bib-0057] Lee, M. , & Jones, D. (2014). Landslide risk assessment (2nd ed.). London: Institution of Civil Engineers.

[gh2190-bib-0058] Li, Z. , Nadim, F. , Huang, H. , Uzielli, M. , & Lacasse, S. (2010). Quantitative vulnerability estimation for scenario‐based landslide hazards. Landslides, 7(2), 125–134. 10.1007/s10346-009-0190-3

[gh2190-bib-0059] Lin, Q. , Wang, Y. , Liu, T. , Zhu, Y. , & Sui, Q. (2017). The vulnerability of people to landslides: A case study on the relationship between casualties and volume of landslides in China. International Journal of Environmental Research and Public Health, 14(2), 212 10.3390/ijerph14020212 PMC5334766 28230810

[gh2190-bib-0060] Marsh, D. , & Schroeder, D. (2002). The positive deviance approach to improve health outcomes: Experience and evidence from the field—Preface. Food and Nutrition Bulletin, 23(4_suppl2), 3–6. 10.1177/15648265020234S201 12503225

[gh2190-bib-0061] Massey, C. , Thomas, K‐L. , King, A. , Singeisen, C. , Taig, T. , & Horspool, N. (2019). SLIDE (Wellington): Vulnerability of dwellings to landslides (Project No. 16/SP740). GNS Science report; 2018/17.

[gh2190-bib-0062] McDougall, S. , McKinnon, M. , & Hungr, O. (2012). Developments in landslide runout prediction In Clague J. & Stead D. (Eds.), Landslides: Types, Mechanisms and Modelling (Chap. 16, pp. 187–195). Cambridge: Cambridge University Press 10.1017/CBO9780511740367.017

[gh2190-bib-0063] McPhee, J. (1989). The control of nature. New York: Farrar, Straus and Giroux.

[gh2190-bib-0064] Memchoubi, P. , Loyi, M. , & Nabachandra, H. (2012). Landslide fatalities: A study of six cases. Journal of Indian Academy of Forensic Medicine, 34, 182–184.

[gh2190-bib-0065] Merchant, R. , Leigh, J. , & Lurie, N. (2010). Health care volunteers and disaster response—First, be prepared. The New England Journal of Medicine, 362(10), 872–873. 10.1056/NEJMp1001737 20181966

[gh2190-bib-0066] Metivier‐Hart, D. (2017). A silent stillness—Buried alive: One woman's remarkable story of survival, hope and rescue; the last survivor of the La Conchita landslide. Xlibris.

[gh2190-bib-0067] Michael‐Leiba, M. , Baynes, F. , Scott, G. , & Granger, K. (2005). Quantitative landslide risk assessment of Cairns, Australia In Glade T., Anderson M., Crozier M. (Eds.), Landslide Hazard and Risk (Chap. 21, pp. 621–642). West Sussex: John Wiley & Sons 10.1002/9780470012659.ch21

[gh2190-bib-0068] Milanesi, L. , Pilotti, M. , & Ranzi, R. (2015). A conceptual model of people's vulnerability to floods. Water Resources Research, 51, 182–197. 10.1002/2014WR016172

[gh2190-bib-0069] Norris, F. , Murphy, A. , Baker, C. , & Perilla, J. (2004). Postdisaster PTSD over four waves of a panel study of Mexico's 1999 flood. Journal of Traumatic Stress, 17(4), 283–292. 10.1023/B:JOTS.0000038476.87634.9b 15462535

[gh2190-bib-0070] Papathoma‐Kohle, M. (2016). Vulnerability curves vs. vulnerability indicators: Application of an indicator‐based methodology for debris‐flow hazards. Natural Hazards and Earth System Sciences, 16(8), 1771–1790. 10.5194/nhess-16-1771-2016

[gh2190-bib-0071] Papathoma‐Kohle, M. , Keiler, M. , Totschnig, R. , & Glade, T. (2012). Improvement of vulnerability curves using data from extreme events: Debris flow event in South Tyrol. Natural Hazards, 64(3), 2083–2105. 10.1007/s11069-012-0105-9

[gh2190-bib-0072] Papathoma‐Kohle, M. , Zischg, A. , Fuchs, S. , Glade, T. , & Keiler, M. (2015). Loss estimation for landslides in mountain areas—An integrated toolbox for vulnerability assessment and damage documentation. Environmental Modelling & Software, 63, 156–169. 10.1016/j.envsoft.2014.10.003

[gh2190-bib-0073] Park, Y. , Pradhan, A. , Kim, U. , Kim, Y.‐T. , & Kim, S. (2016). Development and application of urban landslide vulnerability assessment methodology reflecting social and economic variables. Advances in Meteorology, 2016, 1–13. 10.1155/2016/4572498

[gh2190-bib-0074] Pereira, B. , Morales, W. , Cardoso, R. , Fiorelli, R. , Fraga, G. , & Briggs, S. (2013). Lessons learned from a landslide catastrophe in Rio de Janeiro, Brazil. American Journal of Disaster Medicine, 8(4), 253–258. 10.5055/ajdm.2013.0131 24481889

[gh2190-bib-0075] Pereira, S. , Zezere, J. , Quaresma, I. , Santos, P. , & Santos, M. (2016). Mortality patterns of hydro‐geomorphologic disasters. Risk Analysis, 36(6), 1188–1210. 10.1111/risa.12516 26616470

[gh2190-bib-0076] Petley, D. (2010). Landslide hazards In Alcantara‐Ayala I. & Goudie A. (Eds.), Geomorphological hazards and disaster prevention (Chap. 6, pp. 63–73). Cambridge: Cambridge U. Press 10.1017/CBO9780511807527.006

[gh2190-bib-0077] Pita, G. , Pinelli, J‐P ., Gurley, K. , Weekes, J. , Mitrain‐Reiser, J. (2011). Wind vulnerability curves for low‐rise commercial‐residential buildings in the Florida public hurricane loss model. First International Symposium on Uncertainty Modelling and Analysis and Management and Fifth International Symposium on Uncertainty Modelling and Analysis. 10.1061/41170(400)75

[gh2190-bib-0078] Pollock, W. (2020). A framework for regional scale quantitative landslide risk analysis. (*doctoral dissertation*). Seattle: University of Washington 10.1016/j.mex.2025.103218 PMC11938156 40144140

[gh2190-bib-0079] Pollock, W. , & Wartman, J. (2020). Human vulnerability to landslides: Fatality dataset. DesignSafe‐CI. 10.17603/ds2-hv13-ra52 PMC7567151 33094206

[gh2190-bib-0080] Prieto, J. , Journeay, M. , Acevedo, A. , Arbalaez, J. , & Ulmi, M. (2018). Development of structural debris flow fragility curves (debris flow buildings resistance) using momentum flux rate as a hazard parameter. Engineering Geology, 239, 144–157. 10.1016/j.enggeo.2018.03.014

[gh2190-bib-0081] Quan Luna, B. , Blahut, J. , van Westen, C. , Sterlacchini, S. , van Asch, T. , & Akbas, O. (2011). The application of numerical debris flow modelling for the generation of physical vulnerability curves. Natural Hazards and Earth System Sciences, 11(7), 2047–2060. 10.5194/nhess-11-2047-2011

[gh2190-bib-0082] Reese, S. , Cousins, W. , Power, W. , Palmer, N. , Tejakusuma, I. , & Nugrahadi, S. (2007). Tsunami vulnerability of buildings and people in South Java—Field observations after the July 2006 Java tsunami. Natural Hazards and Earth System Sciences, 7(5), 573–589. 10.5194/nhess-7-573-2007

[gh2190-bib-0083] Safeland . (2011). Physical vulnerability of elements at risk to landslides: Methodology for evaluation, fragility curves and damage states for buildings and lifelines. Safeland Deliverable, 2, 5 Available at:. https://cordis.europa.eu/project/id/226479/reporting

[gh2190-bib-0084] Salvati, P. , Petrucci, O. , Rossi, M. , Bianchi, C. , Pasqua, A. , & Guzzetti, F. (2018). Gender, age and circumstances analysis of flood and landslide fatalities in Italy. Science of the Total Environment, 610–611, 867–879. 10.1016/j.scitotenv.2017.08.064 28826124

[gh2190-bib-0085] Sanchez, C. , Lee, T.‐S. , Young, S. , Batts, D. , Benjamin, J. , & Malilay, J. (2009). Risk factors for mortality during the 2002 landslides in Chuuk, Federated States of Micronesia. Disasters, 33(4), 705–720. 10.1111/j.1467-7717.2009.01105.x 19459918

[gh2190-bib-0086] Santa Barbara County Sheriff's Office (SBCSO) . (2018). “Names released of Montecito residents fatally injured during flood incident.” Available at: https://www.sbsheriff.org/names‐released‐montecito‐residents‐fatally‐injured‐flood‐incident/

[gh2190-bib-0087] Santi, P. , Hewitt, K. , VanDine, D. , & Cruz, E. (2011). Debris‐flow impact, vulnerability, and response. Natural Hazards, 56(1), 371–402. 10.1007/s11069-010-9576-8

[gh2190-bib-0088] Sattler, D. , Kaiser, C. , & Hittner, J. (2000). Disaster preparedness: Relationships among prior experience, personal characteristics, and distress. Journal of Applied Social Psychology, 30, 1396–1420. 10.1111/j.1559-1816.2000.tb02527.x

[gh2190-bib-0089] Schilling, S. (2014). Laharz_py: GIS tools for automated mapping of lahar inundation hazard zones. USGS Open‐File Report 2014–1073. Reston, VA: U.S. Geological Survey.

[gh2190-bib-0090] Schuster, R. , Highland, L. (2001). Socioeconomic and environmental impacts of landslides in the western hemisphere. U.S. Geological Survey Open‐File Report 01–276.

[gh2190-bib-0091] Sheeju, P. , Hussain, S. , & Balaram, N. (2019). Patterns of injuries in victims of landslide. Journal of Medical Science And clinical Research, 7(10), 123–127. 10.18535/jmscr/v7i10.24

[gh2190-bib-0092] Snohomish County Medical Examiner's Office (MEO) . (2014). “Media update as of July 23, 2014 at 9:00 AM.” https://snohomishcountywa.gov/ArchiveCenter/ViewFile/Item/3913

[gh2190-bib-0093] Spence, R. , So, E. , Jenny, S. , Castella, H. , Ewald, M. , & Booth, E. (2008). The Global Earthquake Vulnerability Estimation System (GEVES): An approach for earthquake risk assessment for insurance applications. Bulletin of Earthquake Engineering, 6(3), 463–483. 10.1007/s10518-008-9072-7

[gh2190-bib-0094] Taig, T. , Massey, C. , Taig, M. , Becker, J. , Heron, D . (2015). Survey of Port Hills red zone residents' experience following the 22 February 2011 earthquake. GNS Science Report 2015/09. GNS Science.

[gh2190-bib-0095] Totschnig, R. , & Fuchs, S. (2013). Mountain torrents: Quantifying vulnerability and assessing uncertainties. Engineering Geology, 155, 31–44. 10.1016/j.enggeo.2012.12.019 27087696 PMC4819033

[gh2190-bib-0096] Totschnig, R. , Sedlacek, W. , & Fuchs, S. (2011). A quantitative vulnerability function for fluvial sediment transport. Natural Hazards, 58(2), 681–703. 10.1007/s11069-010-9623-5

[gh2190-bib-0097] Tsao, T‐C ., Hsu, W‐K ., Cheng, C‐T ., Lo, W‐C ., Chen, C‐Y ., Chang, Y‐L. , & Ju, J‐P . (2010). A preliminary study of debris flow risk estimation and management in Taiwan. In: S‐C. Chen (*Ed*.) Internationales Symposion Interpraevent in the Pacific rim: Taipei.

[gh2190-bib-0098] UNISDR (2009). Chapter 2: Global disaster risk: Patterns, trends and drivers. In: *Global Assessment Report on Disaster Risk Reduction* . https://www.preventionweb.net/english/hyogo/gar/report/index.php?id=9413

[gh2190-bib-0099] United Nations . (2019). World Population Prospects 2019. United Nations, Department of Economic and Social Affairs, Population Division. Online Edition: https://population.un.org/wpp/

[gh2190-bib-0100] United Nations . (2020). World economic situation and prospects. New York: United Nations. https://www.un.org/development/desa/dpad/publication/world‐economic‐situation‐and‐prospects‐2020/

[gh2190-bib-0101] Uzielli, M. , Nadim, F. , Lacasse, S. , & Kaynia, A. (2008). A conceptual framework for quantitative estimation of physical vulnerability to landslides. Engineering Geology, 102(3–4), 251–256. 10.1016/j.enggeo.2008.03.011

[gh2190-bib-0102] Vollmer, J. , Schulze, P. , & Chebra, J. (2005). The American master bedroom: Its changing location and significance to the family. Journal of Interior Design, 31(1), 1–13. 10.1111/j.1939-1668.2006.tb00413.x

[gh2190-bib-0104] Wartman, J. , Montgomery, D. , Anderson, S. , Keaton, J. , Benoit, J. , dela Chapelle, J. , & Gilbert, R. (2016). The 22 March 2014 Oso landslide, Washington, USA. Geomorphology, 253, 275–288. 10.1016/j.geomorph.2015.10.022

[gh2190-bib-0105] Washington Geological Survey (WGS) . (2017). Landslide hazards in Washington State. Washington Geological Survey Fact Sheet. https://www.dnr.wa.gov/publications/ger_fs_landslide_hazards.pdf

[gh2190-bib-0106] Washington Geological Survey (WGS) and Oregon Department of Geology and Mineral Industries (DOGAMI) . (2015). A homeowner's guide to landslides for Washington and Oregon. https://www.dnr.wa.gov/publications/ger_homeowners_guide_landslides.pdf

[gh2190-bib-0107] Winter, M. , Smith, J. , Fotopoulou, S. , Pitilakis, K. , Mavrouli, O. , Corominas, J. , & Agyroudis, S. (2013). The physical vulnerability of roads to debris flow. 18^th^ International Conference on Soil Mechanics and Geotechnical Engineering: Challenges and innovations in geotechnics, Paris.

[gh2190-bib-0108] Wishik, S. , & Van der Vynckt, S. (1976). The use of nutritional “positive deviants” to identify approaches for modification of dietary practices. American Journal of Public Health, 66(1), 38–42. 10.2105/ajph.66.1.38 1247135 PMC1653353

[gh2190-bib-0109] Wisner, B. , Blaikie, P. , Cannon, T. , & Davis, I. (2004). At risk: Natural hazards, people's vulnerability and disasters. New York: Routledge.

[gh2190-bib-0110] Wray, J. (1972). Can we learn from successful mothers? The Journal of Tropical Pediatrics and Environmental Child Health, 18(4), 279 10.1093/tropej/18.4.279 4495129

[gh2190-bib-0111] Yang, P. , Yen, C.‐F. , Tang, T.‐C. , Chen, C.‐S. , Yang, R.‐C. , Huang, M.‐S. , Jong, Y.‐J. , & Yu, H.‐S. (2011). Posttraumatic stress disorder in adolescents after Typhoon Morakot‐associated mudslides. Journal of Anxiety Disorders, 25(3), 362–368. 10.1016/j.janxdis.2010.10.010 21126851

[gh2190-bib-0112] Zahran, S. , Peek, L. , & Brody, S. (2008). Youth mortality by forces of nature. Children, Youth and Environments, 18, 371–388.

[gh2190-bib-0113] Zhang, J. , Gurung, D. , Liu, R. , Murthy, M. , & Su, F. (2015). Abe Barek landslide and landslide susceptibility assessment in Badakhshan Province, Afghanistan. Landslides, 12(3), 597–609. 10.1007/s10346-015-0558-5

[gh2190-bib-0114] Zhang, S. , Zhang, L. , Li, X. , & Xu, Q. (2018). Physical vulnerability models or assessing building damage by debris flows. Engineering Geology, 247, 145–158. 10.1016/j.enggeo.2018.10.017

